# Differential Incidence of Malaria in Neighboring Villages in a High-Transmission Setting of Southern Mali

**DOI:** 10.4269/ajtmh.21-0788

**Published:** 2022-02-28

**Authors:** Bakary Fofana, Shannon Takala-Harrison, Amed Ouattara, Issaka Sagara, Amadou H. Togo, Hamadoun Diakité, Mohamed Keita, Kassim Sanogo, Sekou Touré, Ogobara K. Doumbo, Abdoulaye A. Djimde

**Affiliations:** ^1^ Malaria Research and training Center/Department of Epidemiology of Parasitic diseases/ Faculty of Pharmacy and Faculty of Medicine and Odontostomatology/University of Sciences, Techniques and Technologies of Bamako, Mali;; ^2^ Center for Vaccine Development and Global Health, University of Maryland School of Medicine, Baltimore, Maryland

## Abstract

Throughout a phase IIIb/IV efficacy study of repeated treatment with four artemisinin-based combination therapies, significant heterogeneity was found in the number of clinical episodes experienced by individuals during the 2-year follow-up. Several factors, including host, parasite, and environmental factors, may contribute to the differential malaria incidence. We aimed to identify risk factors of malaria incidence in the context of a longitudinal study of the efficacy of different artemisinin-based combination therapy regimens in Bougoula-Hameau, a high-transmission setting in Mali. Risk factors including age, residence, and treatment regimen were compared among individuals experiencing eight or more clinical episodes of malaria (“high-incidence group”) and individuals experiencing up to three clinical episodes (“low-incidence group”). Consistent with the known association between age and malaria risk in high-transmission settings, individuals in the high incidence group were significantly younger than individuals in the low-risk group (mean age, 7.0 years versus 10.6 years, respectively; *t*-test, *P* < 0.0001). Compared with individuals receiving artemether-lumefantrine, those receiving artesunate-amodiaquine had greater odds of being in the high-incidence group (odds ratio [OR], 2.24; 95% CI, 1.03 – 4.83, *P* = 0.041), while individuals receiving dihydroartemisinin-piperaquine had a lower odds of being in high incidence group (OR: 0.30, 95% CI, 0.11–0.85; *P* = 0.024). Individuals residing in the forested areas of Sokourani and Karamogobougou had significantly greater odds of being in the high-incidence group compared with individuals residing in the semi-urban area of Bougoula-Hameau 1 (Karamogobougou: OR, 3.68; 95% CI, 1.46–9.31; *P* = 0.0059; Sokourani: OR, 11.46; 95% CI, 4.49–29.2; *P* < 0.0001). This study highlights the importance of fine-mapping malaria risks even at sub-district levels for targeted and customized interventions.

## INTRODUCTION

The 2020 World Malaria Report estimates that there were 229 million cases and 409,000 deaths from malaria globally in 2019. ^1^ Malaria transmission is characterized by important microgeographic variations, ^2^ often between adjacent villages, households, or families, ^2^^,^ ^3^ and is strongly associated with location. ^3^ Many factors can contribute to this variable risk of exposure and disease, including design of housing, proximity to mosquito breeding sites, poor access to treatment, maternal education, wealth, and other yet-undefined characteristics. ^4^^,^ ^5^ In addition to the heterogeneity in exposure to the parasite, individual responses to infection are also different because of immune status, age, and other factors.

Artemether–lumefantrine and artesunate–amodiaquine are first-line treatments for uncomplicated malaria in many endemic countries, including Mali. Dihydroartemisinin–piperaquine is an alternative, safe, and efficacious first-line artemisinin-based combination therapy (ACT) in Mali. ^6^^,^ ^7^ More recently, artesunate–pyronaridine has been approved for the treatment of uncomplicated malaria and was added to the panel of first-line treatments in some countries. ^1^

Throughout the initial clinical and parasitological evaluation of the effect of repetitive administration of four ACTs on the incidence density of malaria during a 2-year follow-up (the West African Network of Clinical Trial of Antimalarials Drugs [WANECAM] study ^7^^,^ ^8^), we demonstrated that some children experienced a large number of clinical episodes of malaria (8–12 episodes), whereas others experienced very few (three episodes or less). Several factors may account for this observation, including human genetic factors, immune status of the individual, parasite virulence, environmental factors that affect exposure to mosquito bites, and the pharmacokinetic properties of each ACT. In addition, the distribution of parasite mutations within genes implicated in drug resistance may impact patients’ responses to treatment as well as the post-treatment protective effect of a given drug or combination therapy. For example, in a population where wild-type *pfmdr1* and *pfcrt* parasite genotypes predominated (≤ 20% *pfmdr1* 86Y and *pfcrt* 76T mutants, respectively), the combination of artesunate–amodiaquine provided an approximate 2-fold longer protective effect than artemether–lumefantrine. ^9^ Conversely, in populations where mutant parasites were more prevalent (i.e., *pfmdr1* 86Y and *pfcrt* 76T mutant parasites > 80%), artemether–lumefantrine provided up to a 1.5-fold longer post-treatment protective effect than artesunate–amodiaquine. ^9^

The pharmacokinetic and pharmacodynamic properties of ACTs have implications for their public health benefit based on their ability to reduce overall malaria transmission in the community, as well as treat disease, ^10^ and for the evolution of drug resistance. The pharmacokinetic/pharmacodynamic properties of different ACTs are different. Pyronaridine has a terminal half-life of at least 14 days, compared with an approximate 8 to 10 days for amodiaquine, 23 to 33 days of piperaquine, and 4 days for lumefantrine. ^11^ Artesunate has a very short elimination half-life of approximately 1 hour and is metabolized rapidly by esterase-catalyzed hydrolysis and CYP2A6 into its active metabolite dihydroartemisinin. ^12^

In a study evaluating the determinants of the post-treatment protective effect of dihydroartemisinin–piperaquine, the day 7 concentration of piperaquine was found to be the main determinant of treatment failure at some of the study sites. ^12^ The study demonstrated that in individuals receiving dihydroartemisinin–piperaquine, the day 7 piperaquine concentration was only associated with treatment failure in individuals infected with piperaquine-sensitive parasites, but this association did not hold when individuals were infected with resistant parasites. ^12^

In this study, we aimed to identify risk factors of malaria in the context of a longitudinal study of the efficacy of different ACT regimens in Bougoula-Hameau, a high-transmission setting in Mali. We hypothesized that individuals having eight or more episodes of clinical malaria during the 2-year study follow-up (compared with individuals experiencing three episodes or fewer) would differ according to host demographic factors (e.g., age or village), residence, and ACT received during the study.

## METHODS

### Study sites.

**Figure 1.**
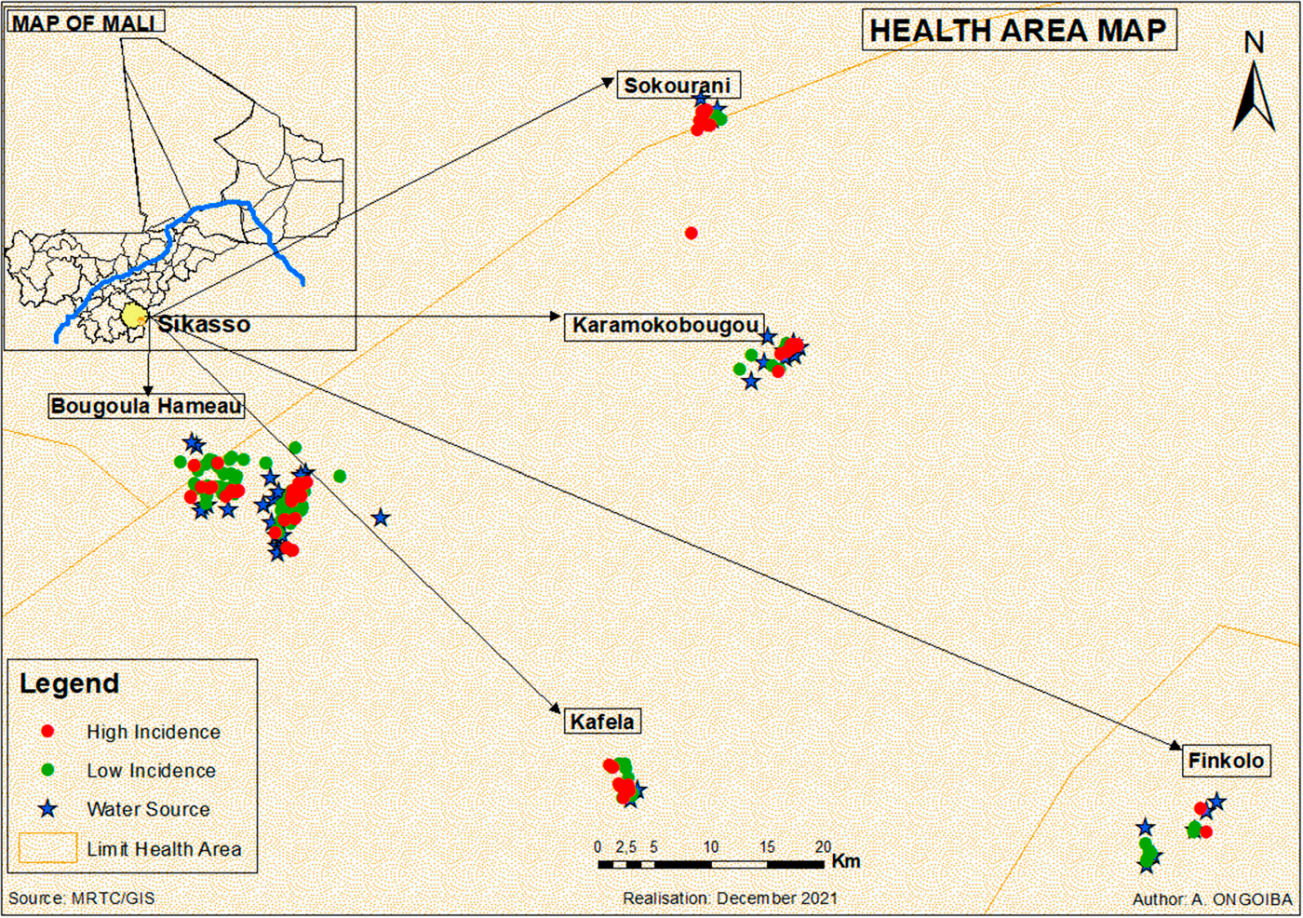
Study geographic locations. This figure appears in color at www.ajtmh.org.

This study was conducted in Bougoula-Hameau, a peri-urban center of approximately 7,000 people located near the city of Sikasso in southern Mali. Bougoula-Hameau and the surrounding villages are located in the Sudanian zone dominated by savanna woodland, with tall grass dotted with trees. The climate is affected by the humid forest zone, with a rainy season of up to 6 months or more. The predominant ethnic groups are Senoufo, Samogo, Mossi, Fulani, and Bambara. The area is rural, with agriculture being the main economic activity. The region receives more rain (average rainfall, 841.4 mm) than any other Malian region, and is known for its fruit and vegetables. *Plasmodium falciparum* is hyperendemic with seasonal peaks during the rainy season. Parasitemia prevalence ranges from 40% to 50% during the dry season (January–May) and 70% to 85% during the rainy season (June–October). ^13^ The site is divided into six geographic clusters or locations identified as Bougoula-Hameau 1, Bougoula-Hameau 2, Sokourani, Karamogobougou, Kafela, and Fincolo. Bougoula-Hameau 1 and Bougoula-Hameau 2 were the main sites of enrollment and are separated by 1 km and a river. Kafela and Fincolo are approximately 3 km apart and are 5 to 7 km from Bougoula-Hameau 1 and Bougoula-Hameau 2. Both Sokourani and Karamogobougou are situated in a forested area near a permanent river within a distance of 7 and 5 km of Bougoula-Hameau 1 and Bougoula-Hameau 2 (Figure 1).

### Study design.

This was a secondary analysis of data collected during a phase IIIb/IV clinical trial of ACTs conducted by the WANECAM study ^14^ conducted from 2012 to 2016 in Bougoula-Hameau and neighboring villages. Patients with uncomplicated malaria were randomized to receive one of the following four ACTs: artesunate–pyronaridine, dihydroartemisinin–piperaquine, artemether–lumefantrine, and artesunate–amodiaquine. After randomization, subjects were treated with the same ACT for all subsequent malaria episodes that occurred during the 2-year follow-up. For each episode, dried blood spots were collected before study drug administration and during follow-up on days 1, 2, 3, 7, 21, 28, 35, 42, and 63. At each visit, a physical examination, including axillary temperature, was performed, and blood was collected for thick smears. Patients were asked to return to the clinic any time they became ill. Any asexual parasite recurrence confirmed by light microscopy after 28 days of treatment initiation was considered a new episode of malaria and was treated using the same ACT administered initially. This study was approved by the Ethics Committee of the Faculty of Medicine and Odontostomatology and the Faculty of Pharmacy at the University of Sciences, Techniques and Technologies of Bamako. Community permission was obtained before the start of the study as described by Diallo et al. ^15^ Written informed consent was obtained appropriately from all participants or from their legal guardians.

Based on our previous report, ^8^ we determined that individuals living in Bougoula-Hameau and neighboring villages—a high-transmission area—have 2.2 episodes of malaria per year. We therefore assumed a mean incidence of four malaria episodes per 2 years per individual. We split our study population into two groups for this secondary analysis based on the mean episode of malaria during the 2-year follow-up. The first group comprised participants with an incidence three or less and the second group included participants with an incidence of 8 or more. Thus, the first group was classified as low incidence and the second group as high incidence. We did not include participants who experienced four to seven episodes of malaria, because we considered their incidence rate to be expected in an area of high-transmission of malaria in Mali. In addition, we did not include participants who withdrew their consent during the study.

### Data analysis.

χ^2^ tests were used to evaluate unadjusted associations between categorical variables (i.e., gender, residence, and treatment regimen) and malaria incidence groups, as defined earlier. *t*-Tests were used to compare unadjusted associations between age (treated as a continuous variable) and malaria incidence. Logistic regression was used to estimate odds ratios (ORs) and 95% CIs of the effect of each variable of interest on malaria incidence classification while adjusting for other variables in the model. Statistical analysis was performed using SAS version 9.4 (SAS Institute, Cary, NC).

## RESULTS

Of the 1,168 patients enrolled in this study, 68 experienced at eight or more episodes of malaria, whereas 602 patients experienced three or fewer episodes of malaria during the 2 years of follow-up, for a total of 670 patients included in the analysis.

Unadjusted associations between malaria incidence and potential risk factors are presented in Table 1. Individuals in the high-incidence group were significantly younger (mean age, 7.0 years) compared with the low-incidence group (10.6 years; *t*-test, *P* < 0.0001). In addition, the distribution of location of residence and treatment arm was significantly different between the susceptible and resistant groups (*P* < 0.0001 for both comparisons, by Fisher’s exact test). There was no significant difference in the distribution of gender between the two incidence groups (*P* = 0.70, Fisher’s exact test), so this variable was not included in the final multivariable logistic regression models.

**Table 1:** Unadjusted associations between malaria incidence and age, gender, location, and treatment arm

Variable	High incidence (*n* = 68)	Low incidence (*n* = 602)	*P* value
Mean age (range), y	7.0 (1.1–17.1)	10.6 (0.7–64.6)	0.0125*
Gender, *n* (%)			
Male	35 (51.5)	291 (48.3)	0.70†
Female	33 (48.5)	311 (51.7)	–
Location, *n *(%)			
Bougoula-Hameau 1	20 (29.5)	247 (41.0)	< 0.0001†
Bougoula-Hameau 2	16 (23.5)	211 (35.1)	–
Fincolo	2 (2.9)	61 (10.1)	–
Kafela	7 (10.3)	41 (6.8)	–
Karamogobougou	10 (14.7)	25 (4.2)	–
Sokourani	13 (19.1)	17 (2.8)	–
Treatment arm, *n *(%)			
Artemether–lumefantrine	16 (23.5)	187 (31.1)	< 0.0001†
Artesunate–pyronaridine	12 (17.7)	110 (18.3)	–
Dihydroartemisinin–piperaquine	33 (48.5)	139 (23.1)	–
Artesunate–amodiaquine	7 (10.3)	166 (27.5)	–

* *t*-Test.

† Fisher’s exact test, two-sided.

After adjusting for all other variables in the model (Table 2, Supplemental Table S1), age showed a significant negative association with malaria incidence, with older individuals having significantly lower odds of being in the high-incidence group (OR, 0.88; 95% CI, 0.82–0.95; *P* = 0.0004). Individuals residing in the forested areas of Sokourani and Karamogobougou had significantly greater odds of being at high incidence compared with individuals residing in Bougoula-Hameau 1 (Karamogobougou: OR, 3.68; 95% CI, 1.46–9.31; *P* = 0.0059; Sokourani: OR, 11.46; 95% CI, 4.49–29.2; *P* < 0.0001). In addition, individuals receiving artesunate–amodiaquine during the study had a greater odds of being in the high-incidence group compared with individuals receiving artemether–lumefantrine (OR, 2.24; 95% CI, 1.03–4.83; *P* = 0.041), whereas individuals receiving dihydroartemisinin–piperaquine had lower odds of being in the high-incidence group compared with individuals receiving artemether–lumefantrine (OR, 0.30; 95% CI, 0.11–0.85; *P* = 0.024). Individuals treated with artesunate–pyronaridine did not show a significant difference in incidence compared with those treated with artemether–lumefantrine (OR, 0.86; 95% CI, 0.36–2.01) during the 2 years of repeated administration of the same ACT.

**Table 2:** Adjusted associations between malaria incidence and potential risk factors

Variable	OR (95% CI)	*P* value
Age	0.88 (0.82–0.95)	0.0004
Location		
Bougoula-Hameau 1	Ref	–
Bougoula-Hameau 2	1.04 (0.52–2.11)	0.91
Fincolo	0.30 (0.07–1.38)	0.12
Kafela	1.98 (0.75–5.20)	0.17
Karamogobougou	3.68 (1.46–9.31)	0.0059
Sokourani	11.46 (4.49–29.2)	< 0.0001
Treatment arm		
Artemether–lumefantrine	Ref	–
Artesunate–pyronaridine	0.86 (0.36–2.01)	0.72
Dihydroartemisinin–piperaquine	0.30 (0.11–0.85)	0.024
Artesunate–amodiaquine	2.24 (1.03–4.83)	0.041

OR = odds ratio; Ref = reference.

## DISCUSSION

Using data collected as part of a phase IIIb/IV study of four ACTs in Mali, in which individuals were treated with the same ACT for any malaria episode for 2 years, we compared patient characteristics between 68 individuals with a high incidence of malaria (high incidence), experiencing eight or more malaria clinical episodes during the 2-year follow-up, and 602 individuals who had a lower incidence (i.e., low incidence), experiencing three or fewer malaria clinical episodes during the 2-year follow-up. Consistent with the known association between age and malaria risk in high-transmission settings, ^16^^,^ ^17^ individuals in the high-incidence group were significantly younger than individuals in the low-incidence group. We also observed significant associations between malaria incidence and village of residence and treatment regimen. This information provides important insights into factors that can be used to stratify malaria incidence in similar high-transmission settings, and can be used to inform malaria control and treatment policies.

In our study, we observed that individuals residing in the two villages in a forested area near a permanent river were significantly more likely to be in the high-incidence group, despite these villages being within 5 to 7 km of the reference village and major population center at Bougoula-Hameau 1, because they have greater exposure. This observation highlights the heterogeneity of malaria transmission, even across small geographic areas, and the impact of microclimate differences (e.g., proximity to vector breeding habitats), on the level of malaria transmission. Spatial heterogeneity in malaria risk was also observed farther north in Bandiagara, Mali, where Coulibaly et al. ^2^ demonstrated high- and low-risk spatial clusters of malaria disease, which corresponded to potential mosquito breeding sites. Likewise, in Kenya, Midega et al. ^18^ demonstrated that wind direction and proximity to larval sites were correlated with malaria risk.

The association between age and malaria risk in high-transmission settings such as Mali has been well documented ^19^^,^ ^20^ and is thought to reflect increased lifetime exposure and greater acquired immunity in older individuals. ^21^ Although immune status may explain, in part, some difference in malaria incidence among individuals, other human genetic factors, including certain hemoglobinopathies or mutations affected drug metabolism, may also contribute to the differential incidence of malaria. Usually, individuals living within the same area are more likely to be related, although populations are becoming more admixed with increased migration to urban areas. Therefore, further investigation of genetic factors between matched high-incidence and low-incidence pairs may elucidate whether host genetic factors are contributing to increased malaria incidence in certain individuals.

In our study, individuals receiving artesunate–amodiaquine had significantly greater odds of being in the high-incidence group compared with individuals receiving artemether–lumefantrine. Amodiaquine is metabolized primarily in the liver by CYP2C8 to its biologically active metabolite desethylamodiaquine, ^22^ and is slowly eliminated and detectable in plasma and blood for up to 1 month after drug administration. ^23^ This result was unexpected, because the terminal half-life of the active metabolite of amodiaquine—N-desethyl amodiaquine—is longer (2.5–18.2 days in adults) ^24^ than that of lumefantrine (2–3 days in healthy volunteers and 4–6 days in patients with *Plasmodium falciparum* malaria). ^25^ However, there is evidence that the post-treatment protective effect of artemether–lumefantrine and artesunate–amodiaquine can be affected by parasite mutations associated with reduced drug sensitivity. ^26^ For example, the presence of the *pfmdr1* 86Y and 1246Y mutations and the *pfcrt* 76T mutation (associated with chloroquine resistance) have been shown to result in reduced sensitivity to amodiaquine, but increased sensitivity to lumefantrine. ^26^ In our study, the increased incidence of malaria in participants receiving artesunate–amodiaquine may reflect a high prevalence of *pfmdr1* and *pfcrt* mutations in the parasite population, as noted in our previous work, conducted in a similar and very close area of our study sites, with 68.1% (*n* = 191) and 75.3% (*n* = 85) of *pfcrt* 76T mutant alleles in 2012 and 2014, respectively, whereas the *pfmdr1* 86Y mutant allele frequency was 26.7% (*n* = 191) and 15.3% (*n* = 85) in 2012 and 2014, respectively. ^27^ Another study, conducted in a different area in Mali with a similar malaria transmission level found a prevalence of 76.7% (*n* = 60) and 18.3% (*n* = 60) of the *pfcrt* 76T and *pfmdr1* 86Y mutant alleles, respectively, in 2016. ^28^

In contrast to artesunate–amodiaquine, in our study, individuals receiving dihydroartemisinin–piperaquine for the 2 years were less likely to be classified as high incidence compared with individuals receiving artemether–lumefantrine. Dihydroartemisinin–piperaquine efficacy in sub-Saharan Africa has been shown to be similar to that of artemether–lumefantrine. ^6^^,^ ^7^ To date, this ACT is used moderately in Mali and we already reported *P. falciparum* infections carrying plasmepsin 2 duplications associated with piperaquine resistance. ^29^ In addition, dihydroartemisinin–piperaquine has been shown to reduce the risk of recurrent malaria compared with artemether–lumefantrine, ^30^^,^ ^31^ and has produced the benefit of reducing *Plasmodium *spp. malaria incidence after repeated treatment. ^7^ These properties and effectiveness of dihydroartemisinin–piperaquine might be explained primarily by the long half-life of piperaquine of approximately 4 weeks, providing longer post-treatment protection than the other ACTs. ^32^

Individuals receiving artesunate–pyronaridine during the 2 years of repeated treatment did not show a significant difference in the odds of being classified as high incidence compared with those receiving artemether–lumefantrine, despite the half-life of pyronaridine being longer than that of lumefantrine, suggesting the impact of other post-treatment protective factors such as human genetic polymorphism or parasite genetic determinants.

Our study demonstrated that host factors (e.g., age), environmental factors (e.g., geographic properties of a region), and treatment policies (e.g., first-line drug treatment) can affect an individual’s risk of malaria. The data also highlight the importance of continued molecular monitoring of mutations within genes that have been shown to modulate susceptibility to multiple drugs (e.g., *pfmdr1* and *pfcrt*), as the distribution of these mutations may affect the efficacy of ACT partner drugs being used in Mali and other high-burden malaria endemic areas. Our results identify a need for evaluating other human genetic factors that may influence the risk for malaria, such as hemoglobinopathies, erythrocyte surface proteins, and immunogenetic factors. Fine-mapping malaria risks, even at sub-district levels, may be required for targeted and customized interventions.

## Supplemental Material


Supplemental materials
