# Supplementary material for: Differential Incidence of Malaria in Neighboring Villages in a High-Transmission Setting of Southern Mali
Source: Am J Trop Med Hyg. 2022 Feb 28;106(4):1209–14. doi: 10.4269/ajtmh.21-0788 (PMC8991365; doi:10.4269/ajtmh.21-0788)
Supplement: Supplementary file 1 [file tpmd210788.SD1.pdf]

Supplemental Table S1: Odds Ratio Estimates between malaria incidence and potential risk factors

Odds Ratio Estimates			
**Effect**	**Point Estimate**	**95% Wald Confidence Limits**	
**Agegrp2**	2.224	0.891	5.550
**Agegrp3**	0.777	0.287	2.107
**AP**	0.827	0.302	2.266
**DHAPip**	0.483	0.147	1.582
**ASAQ**	2.304	0.938	5.661
**KMG_SKN**	7.200	2.859	18.133
**Kafela**	1.869	0.612	5.710
**Finkolo**	0.679	0.127	3.632
